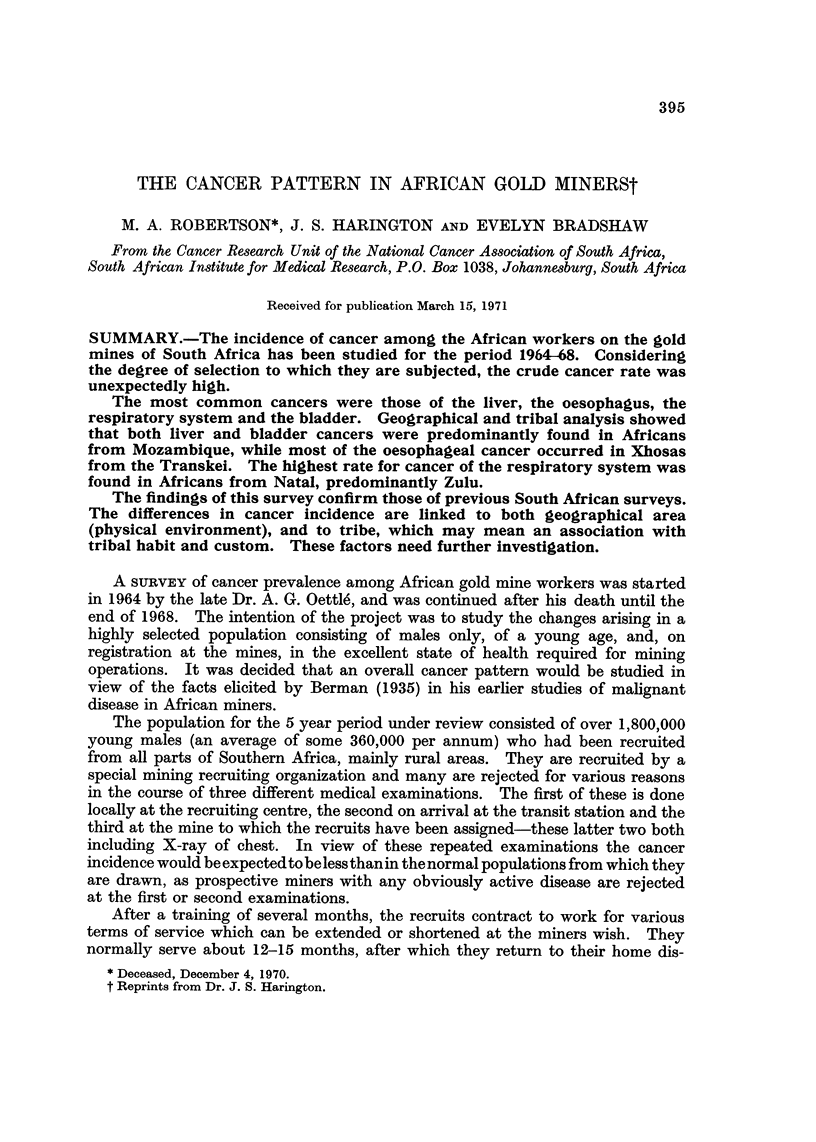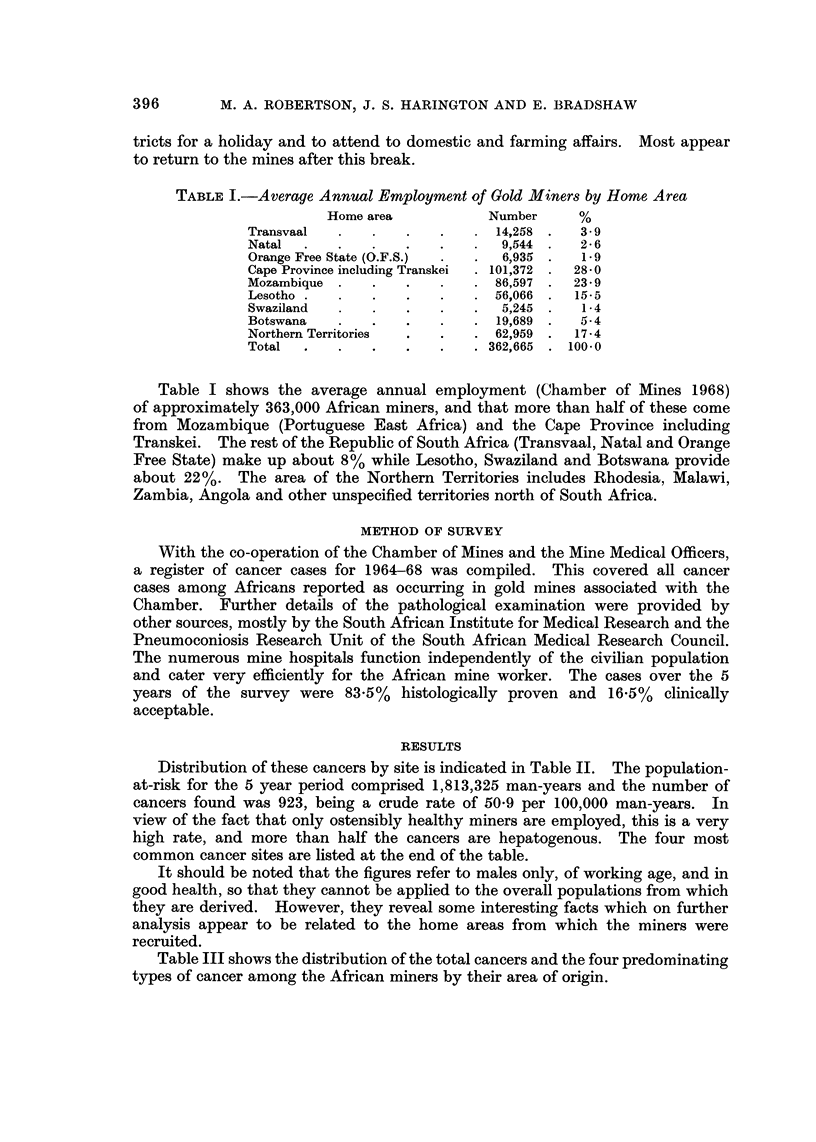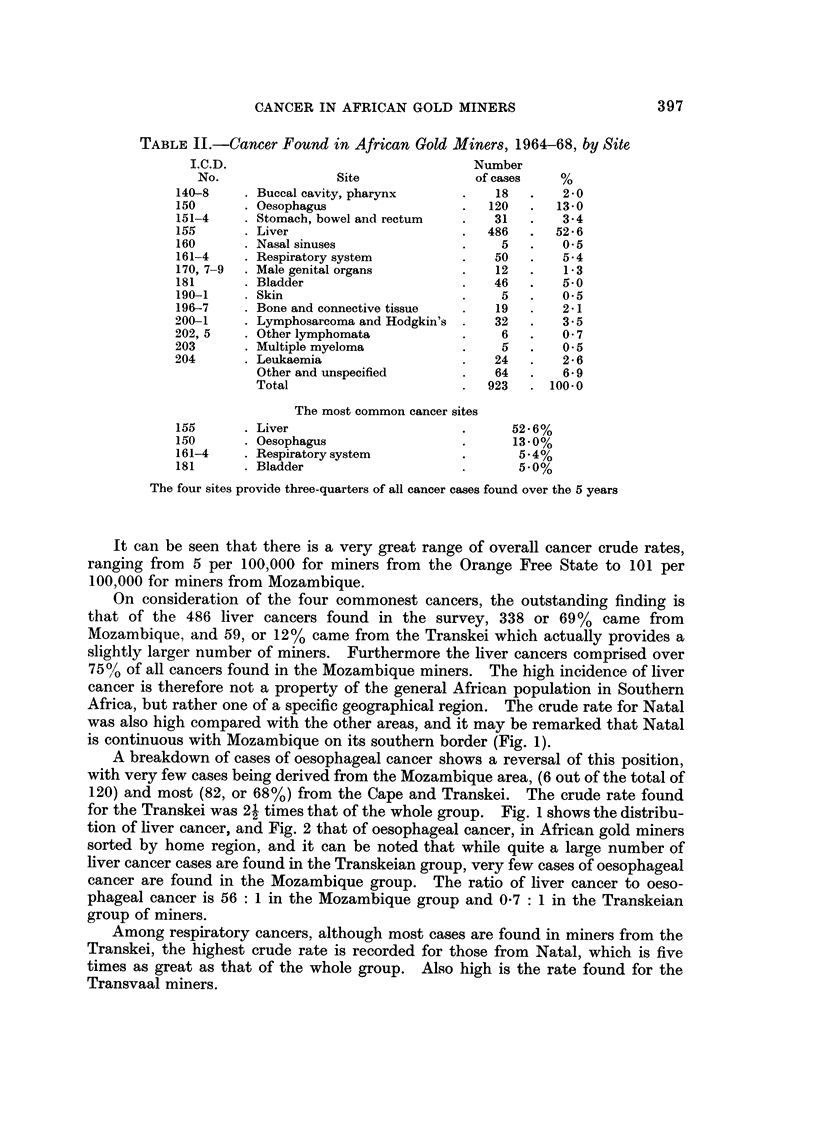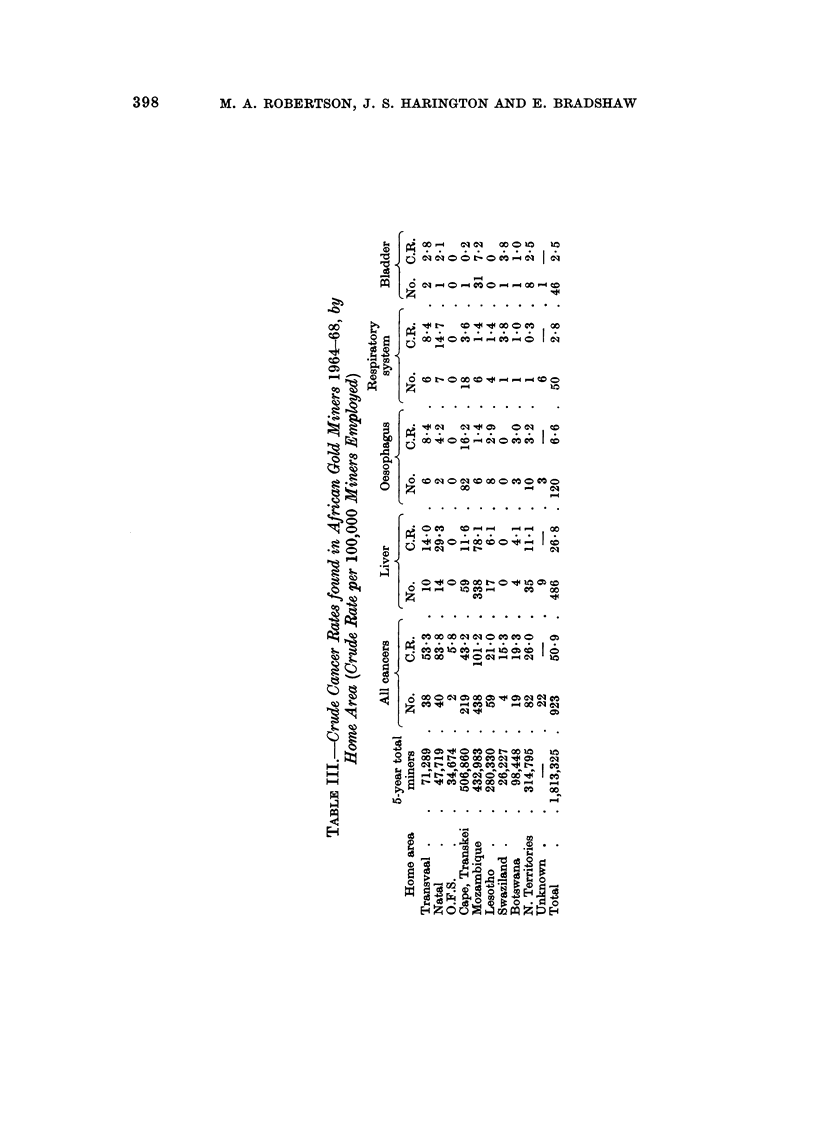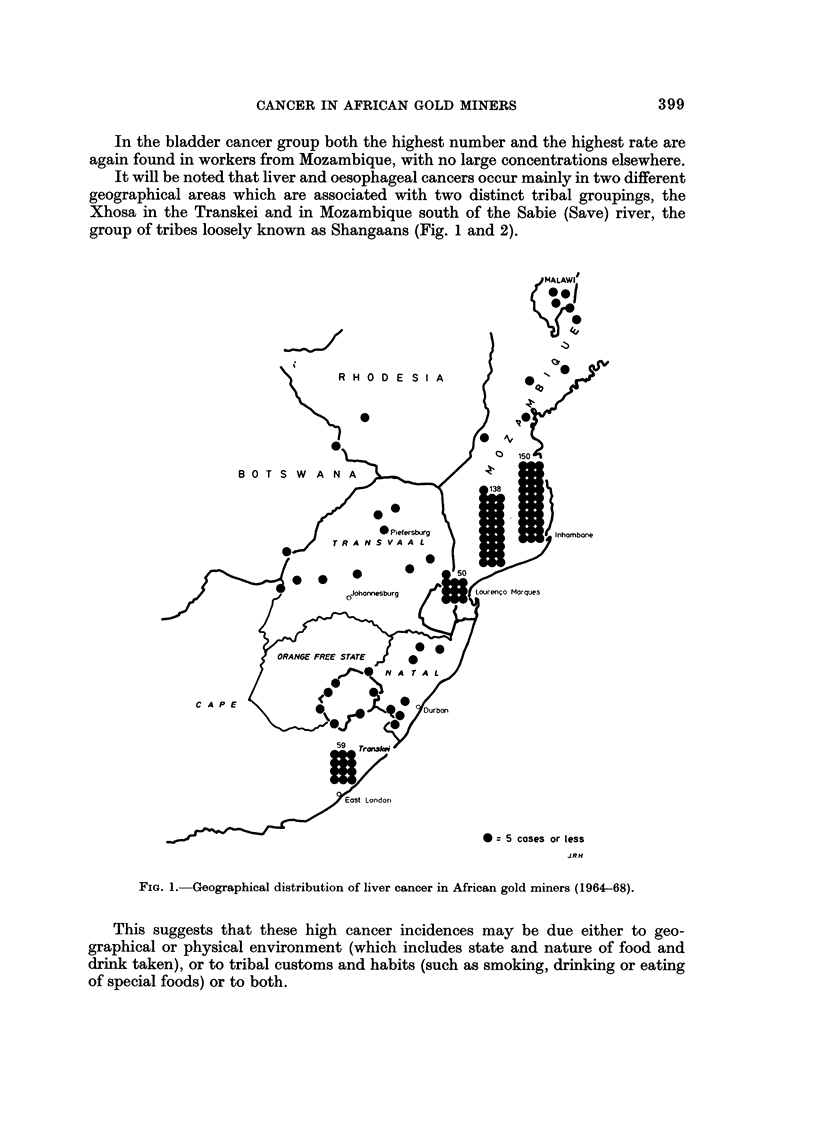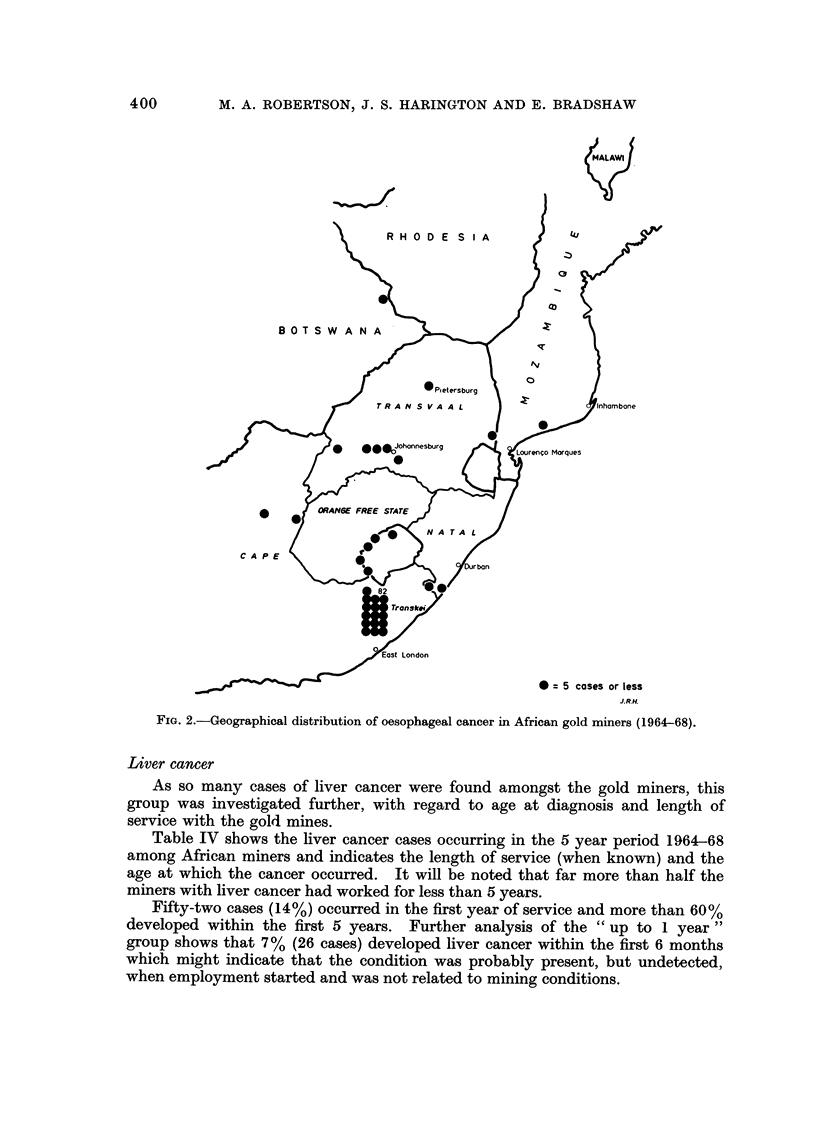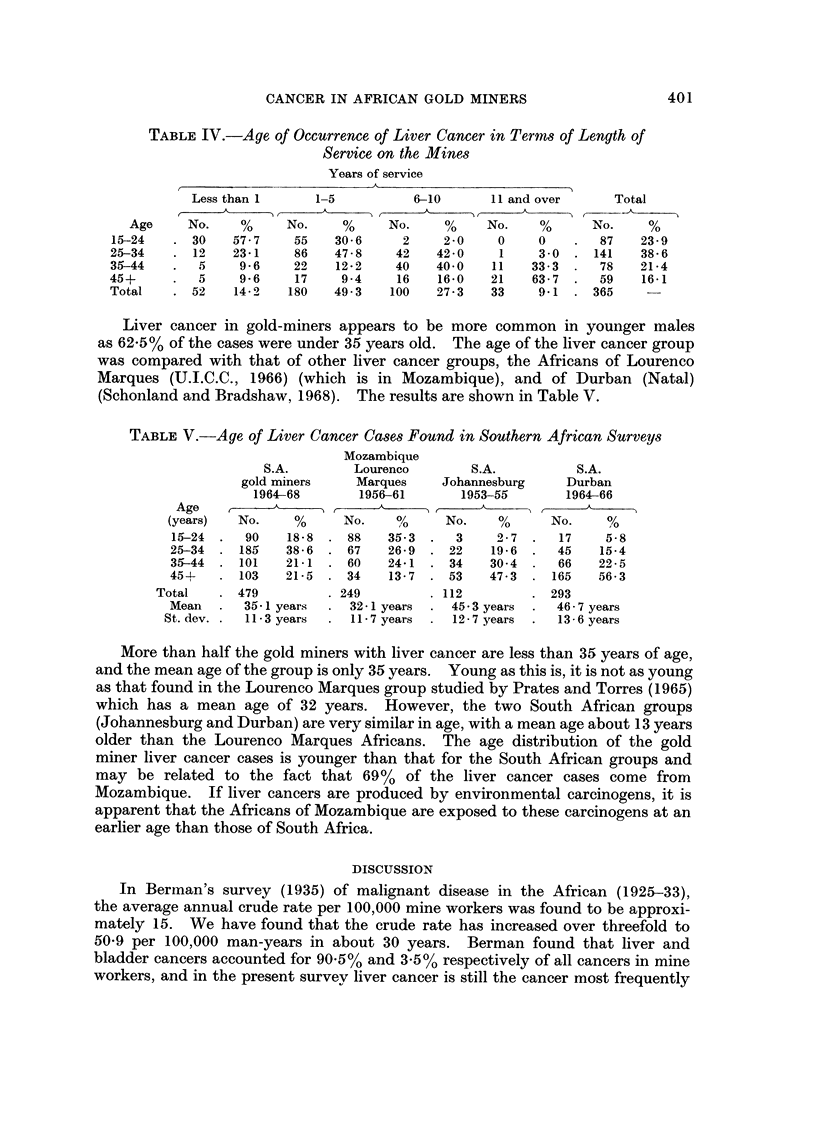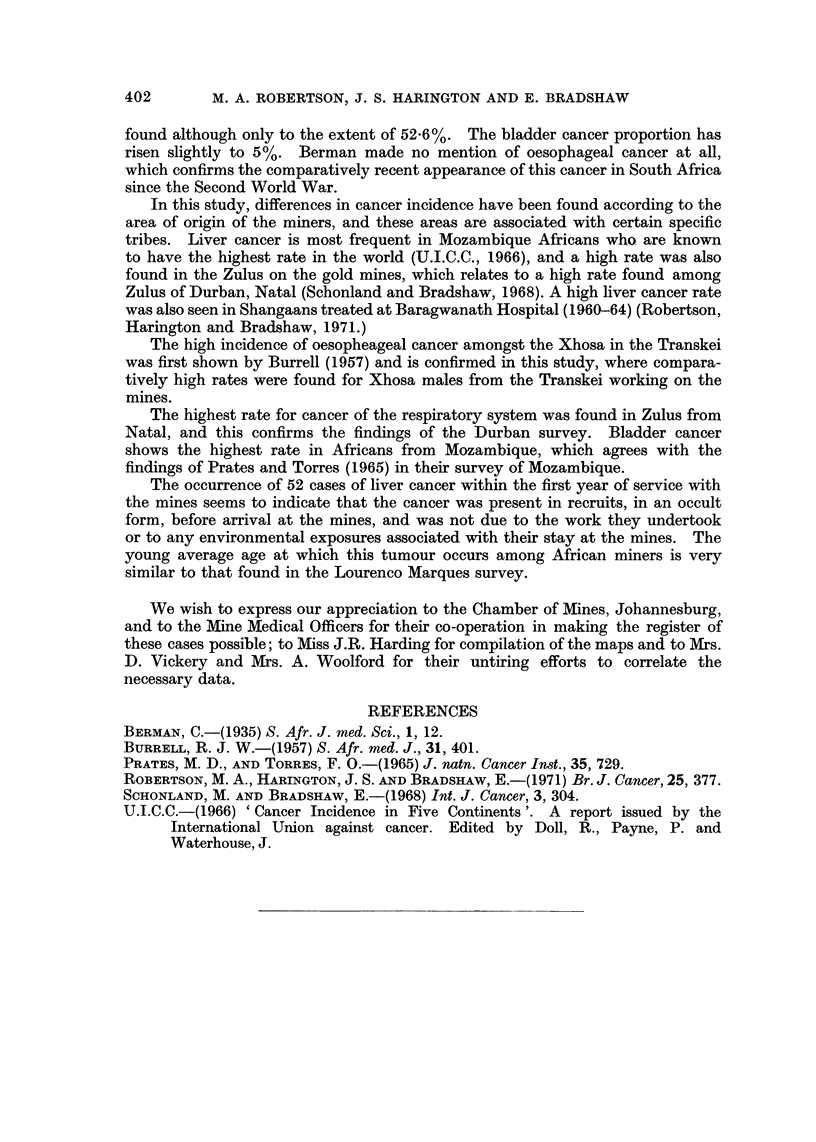# The Cancer Pattern in African Gold Miners†

**DOI:** 10.1038/bjc.1971.50

**Published:** 1971-09

**Authors:** M. A. Robertson, J. S. Harington, Evelyn Bradshaw

## Abstract

The incidence of cancer among the African workers on the gold mines of South Africa has been studied for the period 1964-68. Considering the degree of selection to which they are subjected, the crude cancer rate was unexpectedly high.

The most common cancers were those of the liver, the oesophagus, the respiratory system and the bladder. Geographical and tribal analysis showed that both liver and bladder cancers were predominantly found in Africans from Mozambique, while most of the oesophageal cancer occurred in Xhosas from the Transkei. The highest rate for cancer of the respiratory system was found in Africans from Natal, predominantly Zulu.

The findings of this survey confirm those of previous South African surveys. The differences in cancer incidence are linked to both geographical area (physical environment), and to tribe, which may mean an association with tribal habit and custom. These factors need further investigation.


### THE CANCER PATTERN IN AFRICAN GOLD MINERS†

M. A. ROBERTSON*, J. S. HARINGTON AND EVELYN BRADSHAW

*From the Cancer Research Unit of the National Cancer Association of South Africa, South African Institute for Medical Research, P.O. Box 1038, Johannesburg, South Africa*

Received for publication March 15, 1971

**SUMMARY.—The incidence of cancer among the African workers on the gold mines of South Africa has been studied for the period 1964–68. Considering the degree of selection to which they are subjected, the crude cancer rate was unexpectedly high.**

**The most common cancers were those of the liver, the oesophagus, the respiratory system and the bladder. Geographical and tribal analysis showed that both liver and bladder cancers were predominantly found in Africans from Mozambique, while most of the oesophageal cancer occurred in Xhosas from the Transkei. The highest rate for cancer of the respiratory system was found in Africans from Natal, predominantly Zulu.**

**The findings of this survey confirm those of previous South African surveys. The differences in cancer incidence are linked to both geographical area (physical environment), and to tribe, which may mean an association with tribal habit and custom. These factors need further investigation.**

A SURVEY of cancer prevalence among African gold mine workers was started in 1964 by the late Dr. A. G. Oettlé, and was continued after his death until the end of 1968. The intention of the project was to study the changes arising in a highly selected population consisting of males only, of a young age, and, on registration at the mines, in the excellent state of health required for mining operations. It was decided that an overall cancer pattern would be studied in view of the facts elicited by Berman (1935) in his earlier studies of malignant disease in African miners.

The population for the 5 year period under review consisted of over 1,800,000 young males (an average of some 360,000 per annum) who had been recruited from all parts of Southern Africa, mainly rural areas. They are recruited by a special mining recruiting organization and many are rejected for various reasons in the course of three different medical examinations. The first of these is done locally at the recruiting centre, the second on arrival at the transit station and the third at the mine to which the recruits have been assigned—these latter two both including X-ray of chest. In view of these repeated examinations the cancer incidence would be expected to be less than in the normal populations from which they are drawn, as prospective miners with any obviously active disease are rejected at the first or second examinations.

After a training of several months, the recruits contract to work for various terms of service which can be extended or shortened at the miners wish. They normally serve about 12–15 months, after which they return to their home dis-

* Deceased, December 4, 1970.
† Reprints from Dr. J. S. Harington.

tricts for a holiday and to attend to domestic and farming affairs. Most appear to return to the mines after this break.

TABLE I.—*Average Annual Employment of Gold Miners by Home Area*

| Home area | Number | % |
|---|---|---|
| Transvaal | 14,258 | 3·9 |
| Natal | 9,544 | 2·6 |
| Orange Free State (O.F.S.) | 6,935 | 1·9 |
| Cape Province including Transkei | 101,372 | 28·0 |
| Mozambique | 86,597 | 23·9 |
| Lesotho | 56,066 | 15·5 |
| Swaziland | 5,245 | 1·4 |
| Botswana | 19,689 | 5·4 |
| Northern Territories | 62,959 | 17·4 |
| Total | 362,665 | 100·0 |

Table I shows the average annual employment (Chamber of Mines 1968) of approximately 363,000 African miners, and that more than half of these come from Mozambique (Portuguese East Africa) and the Cape Province including Transkei. The rest of the Republic of South Africa (Transvaal, Natal and Orange Free State) make up about 8% while Lesotho, Swaziland and Botswana provide about 22%. The area of the Northern Territories includes Rhodesia, Malawi, Zambia, Angola and other unspecified territories north of South Africa.

### METHOD OF SURVEY

With the co-operation of the Chamber of Mines and the Mine Medical Officers, a register of cancer cases for 1964–68 was compiled. This covered all cancer cases among Africans reported as occurring in gold mines associated with the Chamber. Further details of the pathological examination were provided by other sources, mostly by the South African Institute for Medical Research and the Pneumoconiosis Research Unit of the South African Medical Research Council. The numerous mine hospitals function independently of the civilian population and cater very efficiently for the African mine worker. The cases over the 5 years of the survey were 83·5% histologically proven and 16·5% clinically acceptable.

### RESULTS

Distribution of these cancers by site is indicated in Table II. The population-at-risk for the 5 year period comprised 1,813,325 man-years and the number of cancers found was 923, being a crude rate of 50·9 per 100,000 man-years. In view of the fact that only ostensibly healthy miners are employed, this is a very high rate, and more than half the cancers are hepatogenous. The four most common cancer sites are listed at the end of the table.

It should be noted that the figures refer to males only, of working age, and in good health, so that they cannot be applied to the overall populations from which they are derived. However, they reveal some interesting facts which on further analysis appear to be related to the home areas from which the miners were recruited.

Table III shows the distribution of the total cancers and the four predominating types of cancer among the African miners by their area of origin.

TABLE II.—*Cancer Found in African Gold Miners*, 1964–68, *by Site*

| I.C.D. No. | Site | Number of cases | % |
|---|---|---|---|
| 140–8 | Buccal cavity, pharynx | 18 | 2·0 |
| 150 | Oesophagus | 120 | 13·0 |
| 151–4 | Stomach, bowel and rectum | 31 | 3·4 |
| 155 | Liver | 486 | 52·6 |
| 160 | Nasal sinuses | 5 | 0·5 |
| 161–4 | Respiratory system | 50 | 5·4 |
| 170, 7–9 | Male genital organs | 12 | 1·3 |
| 181 | Bladder | 46 | 5·0 |
| 190–1 | Skin | 5 | 0·5 |
| 196–7 | Bone and connective tissue | 19 | 2·1 |
| 200–1 | Lymphosarcoma and Hodgkin's | 32 | 3·5 |
| 202, 5 | Other lymphomata | 6 | 0·7 |
| 203 | Multiple myeloma | 5 | 0·5 |
| 204 | Leukaemia | 24 | 2·6 |
| | Other and unspecified | 64 | 6·9 |
| | Total | 923 | 100·0 |

The most common cancer sites

| | | |
|---|---|---|
| 155 | Liver | 52·6% |
| 150 | Oesophagus | 13·0% |
| 161–4 | Respiratory system | 5·4% |
| 181 | Bladder | 5·0% |

The four sites provide three-quarters of all cancer cases found over the 5 years

It can be seen that there is a very great range of overall cancer crude rates, ranging from 5 per 100,000 for miners from the Orange Free State to 101 per 100,000 for miners from Mozambique.

On consideration of the four commonest cancers, the outstanding finding is that of the 486 liver cancers found in the survey, 338 or 69% came from Mozambique, and 59, or 12% came from the Transkei which actually provides a slightly larger number of miners. Furthermore the liver cancers comprised over 75% of all cancers found in the Mozambique miners. The high incidence of liver cancer is therefore not a property of the general African population in Southern Africa, but rather one of a specific geographical region. The crude rate for Natal was also high compared with the other areas, and it may be remarked that Natal is continuous with Mozambique on its southern border (Fig. 1).

A breakdown of cases of oesophageal cancer shows a reversal of this position, with very few cases being derived from the Mozambique area, (6 out of the total of 120) and most (82, or 68%) from the Cape and Transkei. The crude rate found for the Transkei was 2½ times that of the whole group. Fig. 1 shows the distribution of liver cancer, and Fig. 2 that of oesophageal cancer, in African gold miners sorted by home region, and it can be noted that while quite a large number of liver cancer cases are found in the Transkeian group, very few cases of oesophageal cancer are found in the Mozambique group. The ratio of liver cancer to oesophageal cancer is 56 : 1 in the Mozambique group and 0·7 : 1 in the Transkeian group of miners.

Among respiratory cancers, although most cases are found in miners from the Transkei, the highest crude rate is recorded for those from Natal, which is five times as great as that of the whole group. Also high is the rate found for the Transvaal miners.

TABLE III.—*Crude Cancer Rates found in African Gold Miners 1964–68, by Home Area (Crude Rate per 100,000 Miners Employed)*

| Home area | 5-year total miners | All cancers | | Liver | | Oesophagus | | Respiratory system | | Bladder | |
|---|---|---|---|---|---|---|---|---|---|---|---|
| | | No. | C.R. | No. | C.R. | No. | C.R. | No. | C.R. | No. | C.R. |
| Transvaal | 71,289 | 38 | 53·3 | 10 | 14·0 | 6 | 8·4 | 6 | 8·4 | 2 | 2·8 |
| Natal | 47,719 | 40 | 83·8 | 14 | 29·3 | 2 | 4·2 | 7 | 14·7 | 1 | 2·1 |
| O.F.S. | 34,674 | 2 | 5·8 | 0 | 0 | 0 | 0 | 0 | 0 | 0 | 0 |
| Cape, Transkei | 506,860 | 219 | 43·2 | 59 | 11·6 | 82 | 16·2 | 18 | 3·6 | 1 | 0·2 |
| Mozambique | 432,983 | 438 | 101·2 | 338 | 78·1 | 6 | 1·4 | 6 | 1·4 | 31 | 7·2 |
| Lesotho | 280,330 | 59 | 21·0 | 17 | 6·1 | 8 | 2·9 | 4 | 1·4 | 0 | 0 |
| Swaziland | 26,227 | 4 | 15·3 | 0 | 0 | 0 | 0 | 1 | 3·8 | 1 | 3·8 |
| Botswana | 98,448 | 19 | 19·3 | 4 | 4·1 | 3 | 3·0 | 1 | 1·0 | 1 | 1·0 |
| N. Territories | 314,795 | 82 | 26·0 | 35 | 11·1 | 10 | 3·2 | 1 | 0·3 | 8 | 2·5 |
| Unknown | — | 22 | — | 9 | — | 3 | — | 6 | — | 1 | — |
| Total | 1,813,325 | 923 | 50·9 | 486 | 26·8 | 120 | 6·6 | 50 | 2·8 | 46 | 2·5 |

In the bladder cancer group both the highest number and the highest rate are again found in workers from Mozambique, with no large concentrations elsewhere.

It will be noted that liver and oesophageal cancers occur mainly in two different geographical areas which are associated with two distinct tribal groupings, the Xhosa in the Transkei and in Mozambique south of the Sabie (Save) river, the group of tribes loosely known as Shangaans (Fig. 1 and 2).

FIG. 1.—Geographical distribution of liver cancer in African gold miners (1964–68).

This suggests that these high cancer incidences may be due either to geographical or physical environment (which includes state and nature of food and drink taken), or to tribal customs and habits (such as smoking, drinking or eating of special foods) or to both.

FIG. 2.—Geographical distribution of oesophageal cancer in African gold miners (1964–68).

*Liver cancer*

As so many cases of liver cancer were found amongst the gold miners, this group was investigated further, with regard to age at diagnosis and length of service with the gold mines.

Table IV shows the liver cancer cases occurring in the 5 year period 1964–68 among African miners and indicates the length of service (when known) and the age at which the cancer occurred. It will be noted that far more than half the miners with liver cancer had worked for less than 5 years.

Fifty-two cases (14%) occurred in the first year of service and more than 60% developed within the first 5 years. Further analysis of the " up to 1 year " group shows that 7% (26 cases) developed liver cancer within the first 6 months which might indicate that the condition was probably present, but undetected, when employment started and was not related to mining conditions.

TABLE IV.—*Age of Occurrence of Liver Cancer in Terms of Length of Service on the Mines*

| | Years of service | | | | | | | | | |
|---|---|---|---|---|---|---|---|---|---|---|
| | Less than 1 | | 1–5 | | 6–10 | | 11 and over | | Total | |
| Age | No. | % | No. | % | No. | % | No. | % | No. | % |
| 15–24 | 30 | 57·7 | 55 | 30·6 | 2 | 2·0 | 0 | 0 | 87 | 23·9 |
| 25–34 | 12 | 23·1 | 86 | 47·8 | 42 | 42·0 | 1 | 3·0 | 141 | 38·6 |
| 35–44 | 5 | 9·6 | 22 | 12·2 | 40 | 40·0 | 11 | 33·3 | 78 | 21·4 |
| 45+ | 5 | 9·6 | 17 | 9·4 | 16 | 16·0 | 21 | 63·7 | 59 | 16·1 |
| Total | 52 | 14·2 | 180 | 49·3 | 100 | 27·3 | 33 | 9·1 | 365 | — |

Liver cancer in gold-miners appears to be more common in younger males as 62·5% of the cases were under 35 years old. The age of the liver cancer group was compared with that of other liver cancer groups, the Africans of Lourenco Marques (U.I.C.C., 1966) (which is in Mozambique), and of Durban (Natal) (Schonland and Bradshaw, 1968). The results are shown in Table V.

TABLE V.—*Age of Liver Cancer Cases Found in Southern African Surveys*

| Age (years) | S.A. gold miners 1964–68 | | Mozambique Lourenco Marques 1956–61 | | S.A. Johannesburg 1953–55 | | S.A. Durban 1964–66 | |
|---|---|---|---|---|---|---|---|---|
| | No. | % | No. | % | No. | % | No. | % |
| 15–24 | 90 | 18·8 | 88 | 35·3 | 3 | 2·7 | 17 | 5·8 |
| 25–34 | 185 | 38·6 | 67 | 26·9 | 22 | 19·6 | 45 | 15·4 |
| 35–44 | 101 | 21·1 | 60 | 24·1 | 34 | 30·4 | 66 | 22·5 |
| 45+ | 103 | 21·5 | 34 | 13·7 | 53 | 47·3 | 165 | 56·3 |
| Total | 479 | | 249 | | 112 | | 293 | |
| Mean | 35·1 years | | 32·1 years | | 45·3 years | | 46·7 years | |
| St. dev. | 11·3 years | | 11·7 years | | 12·7 years | | 13·6 years | |

More than half the gold miners with liver cancer are less than 35 years of age, and the mean age of the group is only 35 years. Young as this is, it is not as young as that found in the Lourenco Marques group studied by Prates and Torres (1965) which has a mean age of 32 years. However, the two South African groups (Johannesburg and Durban) are very similar in age, with a mean age about 13 years older than the Lourenco Marques Africans. The age distribution of the gold miner liver cancer cases is younger than that for the South African groups and may be related to the fact that 69% of the liver cancer cases come from Mozambique. If liver cancers are produced by environmental carcinogens, it is apparent that the Africans of Mozambique are exposed to these carcinogens at an earlier age than those of South Africa.

### DISCUSSION

In Berman's survey (1935) of malignant disease in the African (1925–33), the average annual crude rate per 100,000 mine workers was found to be approximately 15. We have found that the crude rate has increased over threefold to 50·9 per 100,000 man-years in about 30 years. Berman found that liver and bladder cancers accounted for 90·5% and 3·5% respectively of all cancers in mine workers, and in the present survey liver cancer is still the cancer most frequently found although only to the extent of 52·6%. The bladder cancer proportion has risen slightly to 5%. Berman made no mention of oesophageal cancer at all, which confirms the comparatively recent appearance of this cancer in South Africa since the Second World War.

In this study, differences in cancer incidence have been found according to the area of origin of the miners, and these areas are associated with certain specific tribes. Liver cancer is most frequent in Mozambique Africans who are known to have the highest rate in the world (U.I.C.C., 1966), and a high rate was also found in the Zulus on the gold mines, which relates to a high rate found among Zulus of Durban, Natal (Schonland and Bradshaw, 1968). A high liver cancer rate was also seen in Shangaans treated at Baragwanath Hospital (1960–64) (Robertson, Harington and Bradshaw, 1971.)

The high incidence of oesopheageal cancer amongst the Xhosa in the Transkei was first shown by Burrell (1957) and is confirmed in this study, where comparatively high rates were found for Xhosa males from the Transkei working on the mines.

The highest rate for cancer of the respiratory system was found in Zulus from Natal, and this confirms the findings of the Durban survey. Bladder cancer shows the highest rate in Africans from Mozambique, which agrees with the findings of Prates and Torres (1965) in their survey of Mozambique.

The occurrence of 52 cases of liver cancer within the first year of service with the mines seems to indicate that the cancer was present in recruits, in an occult form, before arrival at the mines, and was not due to the work they undertook or to any environmental exposures associated with their stay at the mines. The young average age at which this tumour occurs among African miners is very similar to that found in the Lourenco Marques survey.

We wish to express our appreciation to the Chamber of Mines, Johannesburg, and to the Mine Medical Officers for their co-operation in making the register of these cases possible; to Miss J.R. Harding for compilation of the maps and to Mrs. D. Vickery and Mrs. A. Woolford for their untiring efforts to correlate the necessary data.

### REFERENCES

Berman, C.—(1935) *S. Afr. J. med. Sci.*, **1**, 12.
Burrell, R. J. W.—(1957) *S. Afr. med. J.*, **31**, 401.
Prates, M. D., and Torres, F. O.—(1965) *J. natn. Cancer Inst.*, **35**, 729.
Robertson, M. A., Harington, J. S. and Bradshaw, E.—(1971) *Br. J. Cancer*, **25**, 377.
Schonland, M. and Bradshaw, E.—(1968) *Int. J. Cancer*, **3**, 304.
U.I.C.C.—(1966) 'Cancer Incidence in Five Continents'. A report issued by the International Union against cancer. Edited by Doll, R., Payne, P. and Waterhouse, J.